# Isolated Adrenocorticotropin Deficiency Associated with Delirium and Takotsubo Cardiomyopathy

**DOI:** 10.1155/2012/580481

**Published:** 2012-12-03

**Authors:** Masanori Murakami, Noriko Matsushita, Rie Arai, Naohiro Takahashi, Ryuki Kawamura, Sayaka Suzuki, Sachio Takekawa, Fumiko Iwashima, Takashi Shibui, Akihiro Hata, Yoshihiro Ogawa, Toshiyuki Horiuchi

**Affiliations:** ^1^Department of Endocrinology and Metabolism, Tokyo Metropolitan Health Medical Treatment Corporation Toshima Hospital, 33-1 Sakae-cho, Itabashi-ku, Tokyo 173-0015, Japan; ^2^Department of Cardiology, Tokyo Metropolitan Health Medical Treatment Corporation Toshima Hospital, 33-1 Sakae-cho, Itabashi-ku, Tokyo 173-0015, Japan; ^3^Department of Psychiatry, Tokyo Metropolitan Health Medical Treatment Corporation Toshima Hospital, 33-1 Sakae-cho, Itabashi-ku, Tokyo 173-0015, Japan; ^4^Department of Molecular Endocrinology and Metabolism, Graduate School of Medical and Dental Sciences, Tokyo Medical and Dental University, 1-5-45 Yushima, Bunkyo-ku, Tokyo 113-8510, Japan

## Abstract

We report a 65-year-old woman with isolated adrenocorticotropic hormone (ACTH) deficiency. The patient was transported to the emergency outpatient department by ambulance complaining of malaise and nausea. Because her laboratory data revealed hyponatremia, we performed endocrinological examinations and diagnosed isolated ACTH deficiency. After admission, she went into a delirious state and suffered from takotsubo cardiomyopathy due to adrenal insufficiency. Replacement therapy with hydrocortisone sufficiently improved her delirium and cardiomyopathy. We conclude that her unstable mental state and myocardial dysfunction were closely related to adrenal insufficiency and suggest that adrenal crisis may cause delirium and Takotsubo cardiomyopathy.

## 1. Introduction

 Isolated adrenocorticotropic hormone (ACTH) deficiency causes secondary hypocortisolism. Major symptoms, which include general malaise, fatigability, anorexia, hypotension, and disturbance of consciousness, are due to hypoglycemia or hyponatremia. Isolated ACTH deficiency is suspected when hypocortisolism is present in addition to the major symptoms already listed. A definite diagnosis requires low or absent serum ACTH or cortisol levels under corticotropin-releasing hormone (CRH) challenge. Although depression-like symptoms are common in hypocortisolism [1], only three cases of delirium with isolated ACTH deficiency have been reported [2–4]. In addition, there were some reports of ACTH deficiency associated with Takotsubo cardiomyopathy [5–9], which is characterized by transient left ventricular apical wall motion abnormality with electrocardiographic changes and minimal myocardial enzymatic release mimicking acute myocardial infarction, but without significant coronary artery disease [10–15]. 

 Here, we report a case of delirium and Takotsubo cardiomyopathy during acute adrenal crisis due to isolated ACTH deficiency.

## 2. Case Report

A 65-year-old woman was admitted to our hospital, complaining of malaise and nausea of one-week duration without preceding infection. She had been in good health until that time except for suffering from Hashimoto's disease, which was treated with thyroid hormone, levothyroxine 75 *μ*g/day in another hospital and was well controlled. She was nulligravida and menopausal at 60 years old. In addition, she had no psychiatric history. 

**Figure 1:**
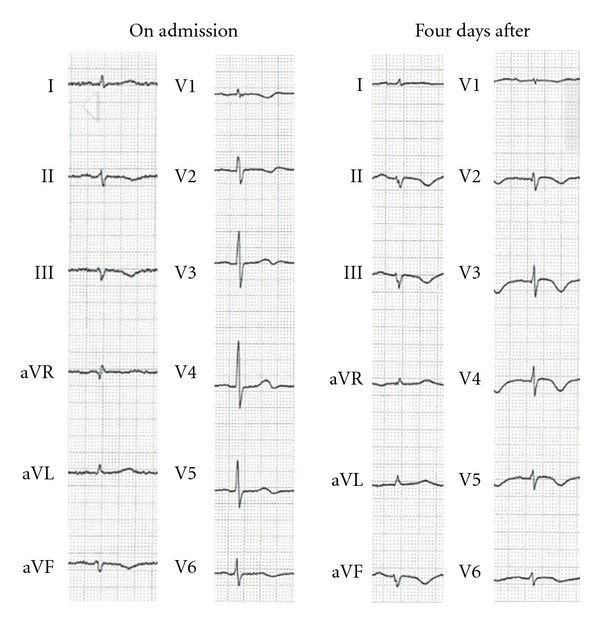
Electrocardiogram changes on admission and four days later while suffering from cardiomyopathy. New inverted T waves were present in the V3-4 leads.

On admission, her body temperature was 35.6°C, blood pressure 157/94 mmHg, and pulse rate 75/min. She exhibited obtundation and her Glasgow Coma Scale was 14 (E4V4M6). Physical examination showed no abnormalities. Her skin and mucosa were free of unusual pigmentation and there were no lack of physiological pigmentation and body hair. Laboratory findings showed only low serum levels of sodium (116 mEq/L, Table 1). On electrocardiogram (ECG), T waves were inverted in leads II, III, V1, and V2 as minor abnormalities (Figure 1). 

**Figure 2:**
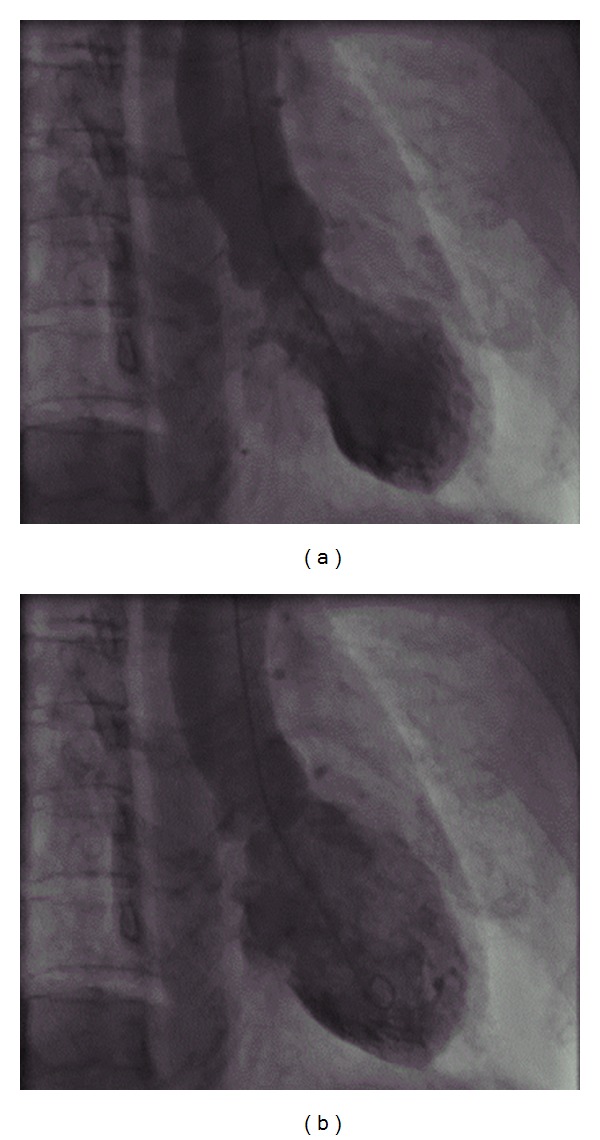
Left ventriculography ((a) systole, (b) diastole) demonstrates akinesis of the apex with systolic ballooning.

 After admission, her consciousness levels were improved for alertness and we tried to treat the hyponatremia by intravenous infusion. However, four days after admission, she presented with paranoia and visual hallucinations. She said, for example, “I was arrested in the hospital,” “The pattern on the walls began to spin,” and “Grass is growing on the window panes.” She was delirious. We consulted psychiatrists who prescribed quetiapine for her delirium. Furthermore, on the same day, she complained of nausea and was pale and sweating. Her blood pressure was 63/48 mmHg, body temperature was 36.0°C, and her SpO_2_ index was unstable (70–90%). She was thought to be in a state of acute circulatory failure. Because the electrocardiogram showed new inverted T waves in leads V2-5 (Figure 1), urgent coronary catherization was performed for possible percutaneous intervention. However, coronary angiography demonstrated no significant coronary stenosis. Left ventriculography showed akinesis of the left ventricular apex with ballooning during systole (Figure 2). Blood examination revealed neither significant hypoglycemia nor electrolyte abnormalities other than hyponatremia (118 mEq/L). The serum levels of creatinine kinase (CK) (603 IU/L), CK-MB (24 IU/L), and troponin I (6.80 ng/mL) were slightly elevated. These findings are consistent with Takotsubo cardiomyopathy. 

 Considering her status of hyponatremia, delirium, and Takotsubo cardiomyopathy, we supposed she could be suffering an acute adrenal crisis and started intravenous administration of 400 mg/day hydrocortisone. On the following day, we confirmed decreased cortisol (2.2 *μ*g/dL, Table 2(a)) and ACTH (8.4 pg/mL) levels and began oral administration of 20 mg/day hydrocortisone. Following the administration of hydrocortisone, the hyponatremia and delirium improved dramatically in 3 days. Her sodium level reached 139 mEq/L and her consciousness level became alert. An echocardiogram showed the left ventricular wall motion abnormalities including apical segment to be also markedly improved by 10 days after steroid administration.

 Endocrine studies showed decreases in serum cortisol and ACTH and reduced urinary secretion of free cortisol. The plasma cortisol level responded slightly after a bolus injection of 0.25 mg ACTH (Table 2(b)). In addition, continuous ACTH-Z stimulation (0.5 mg injected daily for 4 days) produced a gradual but clear increase in the urinary excretion of free cortisol (Table 2(d)). Treatment with ACTH-releasing hormone did not increase the plasma ACTH concentration (Table 2(c)). On the other hand, growth hormone (GH) levels were increased by GH-releasing hormone (Table 2(c)). Although the luteinizing hormone (LH) and follicle stimulating hormone (FSH) levels were slightly increased by LH-releasing hormone, their basal levels were high enough because of her postmenopausal state, and we consider that LH- and FSH-releasing ability was unimpaired (Table 2(c)). We did not perform an infusing TSH-releasing hormone test. Because she was under treatment for Hashimoto's disease involving levothyroxine intake and euthyroid upon admission (Table 2(a)), her hypothalamus function with respect to TSH-releasing ability should not have been impaired. We also did not perform an insulin tolerance test (ITT) because she was elderly and the possibility of severe cardiac involvement was present. Plasma renin activity and plasma aldosterone concentration were normal (Table 2(a)). Therefore, the diagnosis of isolated ACTH deficiency was made. Magnetic resonance imaging (MRI) revealed no pituitary lesions or adjacent lesions.

## 3. Discussion

Adrenal insufficiency was conclusively diagnosed following endocrinological examination based on the markedly decreased plasma cortisol level. The ACTH level was low while the remaining pituitary functions were normal, which led to the diagnosis of isolated ACTH deficiency. Although our patient complained of malaise and nausea for a week prior to admission, the plasma cortisol level responded slightly (<18 *μ*g/mL) after a bolus injection of 0.25 mg ACTH (Table 2(b)). It was considered that she had suffered secondary insufficiency for more than a week.

 Three cases of delirium concurrent with isolated ACTH deficiency have been reported [2–4] (Table 3). Administration of hydrocortisone dramatically improved consciousness in all cases including ours, and this immediate improvement suggests significant links between delirium and adrenal insufficiency. Interestingly, the patient in our case showed paranoid thinking and visual hallucinations. In the previous reports, the patients showed symptoms that included incoherent speech, disorientation, and incontinence [2–4]. We believe our case is the first to describe delirium concurrent with isolated ACTH deficiency, presenting paranoid thinking and visual hallucinations. Considering all reports [2–4] (Table 3), most cases developed delirium with preceding unresolved gastrointestinal symptoms in accordance with hyponatremia in geriatric patients. In addition, they did not have any special psychiatric history. Facing delirium patients having these situations, clinicians should pay attention to adrenal insufficiency as a cause of delirium. 

 Takotsubo cardiomyopathy is now an established syndrome worldwide [16–19], but there are no universally accepted diagnostic criteria for this syndrome. Major clinical characteristics are as follows. First, the symptoms are similar to those of acute myocardial infarction. Second, most cases involve elderly woman. Third, Takotsubo-like left ventricular dysfunction, which extends over more than one coronary artery region, is transient and resolves within several weeks. These findings are consistent with our case. Furthermore, myocardial damage is usually excluded when the CK-MB is <5 or 6% of the total CK activity; in our case, the CK-MB was 4.1% of the total CK activity. The CK elevation may result not from myocardial damage, but rather from the skeletal muscle. Pheochromocytoma can cause myocardial damage unrelated to the coronary arteries [20]. Therefore, although we measured the plasma and urine catecholamine levels, they were within normal limits (Table 2(a)). In addition, computed tomography of the abdomen did not indicate the presence of a mass lesion, which suggests pheochromocytoma; therefore, we ruled out pheochromocytoma.

In our case, immediate replacement therapy with hydrocortisone sufficiently improved her cardiomyopathy. Therefore, we could conclude that her myocardial dysfunction was closely related to adrenal insufficiency. Six cases of reversible left ventricular dysfunction or cardiomyopathy associated with isolated ACTH deficiency have been reported previously [5–9]. Similar to these six cases, intensive stress induced by adrenal insufficiency caused reversible cardiomyopathy in our patient, although the detailed mechanism of left ventricular dysfunction in Takotsubo cardiomyopathy remains unknown [10–17]. According to the report by Ukita et al. [8], they listed all cases of Takotsubo cardiomyopathy with isolated ACTH deficiency [5–7]. Comparing those cases, we found that all cases including ours were female patients although we could not figure out the meanings of the sex difference. Furthermore, though patients with Takotsubo cardiomyopathy often feel chest pain, most of cases with isolated ACTH deficiency did not feel chest pain including ours. Interestingly, Gotyo et al. reported male patient developing Takotsubo cardiomyopathy with idiopathic ACTH deficiency[9]. Their patient also impaired GH secretion, and it was not isolated ACTH deficiency with regard to definition; therefore, they used the word idiopathic ACTH deficiency. 

 To our knowledge, this case represents the first report of both delirium and Takotsubo cardiomyopathy associated with isolated ACTH deficiency. In summary, the present case of a patient suffering from delirium and Takotsubo cardiomyopathy was subsequently diagnosed with adrenal insufficiency induced by isolated ACTH deficiency secondary to a diagnosis of hyponatremia. Both symptoms resolved immediately after the start of steroid replacement therapy with no subsequent relapse; therefore, the unstable mental status and cardiac dysfunction are considered to have been secondary to adrenal insufficiency. Therefore, patients with delirium or Takotsubo cardiomyopathy with no apparent etiology should be examined for adrenal function and begin immediate steroid replacement, if needed.

## Figures and Tables

**Table 1:** Laboratory findings on admission.

Peripheral blood	
White blood cells	4600/*μ*L (3300–9500)
Neutrophils	68.5% (37–80)
Lymphocytes	19.7% (11–50)
Monocytes	10.3% (4–11)
Basophils	0.2% (0–2)
Eosinophils	1.3% (0–8)
Red blood cells	386 × 10^4^/*μ*L (389–501)
Hemoglobin	11.7 g/dL (11.3–15.2)
Hematocrit	30.2% (33.4–46.4)
Platelet	16.2 × 10^4^/*μ*L (14–36)

Serum chemistry	
Sodium	116 mEq/L (136–147)
Potassium	4.5 mEq/L (3.5–5.0)
Chloride	84 mEq/L (95–110)
Glucose	132 mg/dL (70–110)
Urea nitrogen	6.4 mg/dL (7.0–22.0)
Creatinine	0.52 mg/dL (0.40–0.90)
Uric acid	3.1 mg/dL (0.0–7.0)
Total protein	6.1 g/dL (6.3–8.5)
Albumin	3.8 g/dL (3.3–5.1)
Aspartate aminotransferase	33 IU/L (10–38)
Alanine aminotransferase	15 IU/L (6–38)
Lactate dehydrogenase	211 IU/L (115–255)
C-reactive protein	0.3 mg/dL (0.0–0.2)

Figures in parentheses indicate the normal range.

**Table:** (a) Basal levels

ACTH	8.4 pg/mL (7.2–63.3)
Cortisol	2.2 *μ*g/dL (4.0–18.3)
TSH	1.52 *μ*IU/mL (0.55–4.78)
FT3	2.38 pg/mL (2.13–4.07)
FT4	1.89 ng/dL (0.89–1.76)
Plasma renin activity	0.3 ng/mL/hr (0.3–2.9)
Plasma aldosterone	76.8 pg/mL (29.9–159.0)
Urine free cortisol	8.7 *μ*g/day (11.2–80.3)
Urine metanephrine	0.03 mg/day (0.04–0.19)
Urine normetanephrine	0.16 mg/day (0.09–0.33)

Figures in parentheses indicate the normal range.

**Table:** (b) Rapid ACTH test

Time (min)	0	30	60
Cortisol (*μ*g/dL)	5.4	14.1	16.8

**Table:** (c) CRH, LHRH, GRH test

Time (min)	0	30	60	90	120
ACTH (pg/mL)	7.8	14.3	11.7	9.2	8.3
Cortisol (*μ*g/dL)	5.7	6.4	5.7	5.4	5.4
LH (mIU/mL)	19.92	64.31	68.04	66.7	62.75
FSH (mIU/mL)	64.50	75.37	79.86	82.44	86.36
GH (ng/mL)	0.64	8.72	12.3	12.1	4.66

**Table:** (d) Continuous ACTH test

Day	1	2	3	4	5	6	7

Urine free cortisol (*μ*g/day)	5.8	7.0	7.5	99.8	546.0	866.0	2950.0

**Table 3:** Reported cases with isolated ACTH deficiency with delirium.

Case	Age/Sex	Past history	Preceding duration of physical symptoms	Mental symptoms	Normalization of delirium	Steroid hormone therapy	Reference
1	68/M	Stroke	4 months	Disorientation to time and place incontinence	1 day	Cortisone acetate 25 mg/d PO for 10 days 37.5 mg/d PO	[2]
2	74/M	—	1 week	Delirious state	3 days	Hydrocortisone Details unclear	[3]
3	67/M	Cholecystitis	7 weeks	Incoherent speech and conduct incontinence	Details unclear	Hydrocortisone 20 mg/d PO	[4]
4	65/F	Hashimoto's disease	1 week	Paranoia visual hallucinations	3 days	Hydrocortisone 400 mg/d IV for 2 days 20 mg/d PO	Present case

PO: per os, IV: intravenously, and d: day.

## References

[1] Arlt W, Allolio B (2003). Adrenal insufficiency. *The Lancet*.

[2] Fang VS, Jaspan JB (1989). Delirium and neuromuscular symptoms in an elderly man with isolated corticotroph-deficiency syndrome completely reversed with glucocorticoid replacement. *Journal of Clinical Endocrinology and Metabolism*.

[3] Nemoto K, Kawanishi Y, Suzuki H, Mizukami K, Asada T (2007). Isolated adrenocorticotropic hormone deficiency presenting with delirium. *American Journal of Psychiatry*.

[4] Imai H, Matsuishi K, Kitamura N (2009). Isolated adrenocorticotropic hormone deficiency accompanied with delirium. *Psychiatry and Clinical Neurosciences*.

[5] Iga K, Hori K, Gen H (1992). Deep negative T waves associated with reversible left ventricular dysfunction in acute adrenal crisis. *Heart and Vessels*.

[6] Eto K, Koga T, Sakamoto A, Kawazoe N, Sadoshima S, Onoyama K (2000). Adult reversible cardiomyopathy with pituitary adrenal insufficiency caused by empty sella—a case report. *Angiology*.

[7] Sakihara S, Kageyama K, Nigawara T, Kidani Y, Suda T (2007). Ampulla (takotsubo) cardiomyopathy caused by secondary adrenal insufficiency in ACTH isolated deficiency. *Endocrine Journal*.

[8] Ukita C, Miyazaki H, Toyoda N, Kosaki A, Nishikawa M, Iwasaka T (2009). Takotsubo cardiomyopathy during acute adrenal crisis due to isolated adrenocorticotropin deficiency. *Internal Medicine*.

[9] Gotyo N, Kida M, Horiuchi T, Hirata Y (2009). Torsade de pointes associated with recurrent ampulla cardiomyopathy in a patient with idiopathic ACTH deficiency. *Endocrine Journal*.

[10] Dote K, Sato H, Tateishi H, Uchida T, Ishihara M (1991). Myocardial stunning due to simultaneous multivessel coronary spasms: a review of 5 cases. *Journal of Cardiology*.

[11] Kawai S, Suzuki H, Yamaguchi H (2000). Ampulla cardiomyopathy (“Takotusbo” cardiomyopathy) reversible left ventricular dysfunction with ST segment elevation. *Japanese Circulation Journal*.

[12] Tsuchihashi K, Ueshima K, Uchida T (2001). Transient left ventricular apical ballooning without coronary artery stenosis: a novel heart syndrome mimicking acute myocardial infarction. Angina Pectoris-Myocardial Infarction Investigations in Japan. *Journal of the American College of Cardiology*.

[13] Akashi YJ, Nakazawa K, Sakakibara M, Miyake F, Koike H, Sasaka K (2003). The clinical features of takotsubo cardiomyopathy. *QJM*.

[14] Kurisu S, Sato H, Kawagoe T (2002). Tako-tsubo-like left ventricular dysfunction with ST-segment elevation: a novel cardiac syndrome mimicking acute myocardial infarction. *American Heart Journal*.

[15] Abe Y, Kondo M, Matsuoka R, Araki M, Dohyama K, Tanio H (2003). Assessment of clinical features in transient left ventricular apical ballooning. *Journal of the American College of Cardiology*.

[16] Bybee KA, Kara T, Prasad A (2004). Systematic review: transient left ventricular apical ballooning: a syndrome that mimics ST-segment elevation myocardial infarction. *Annals of Internal Medicine*.

[17] Sharkey SW, Shear W, Hodges M, Herzog CA (1998). Reversible myocardial contraction abnormalities in patients with an acute noncardiac illness. *Chest*.

[18] Seth PS, Aurigemma GP, Krasnow JM, Tighe DA, Untereker WJ, Meyer TE (2003). A syndrome of transient left ventricular apical wall motion abnormality in the absence of coronary disease: a perspective from the United States. *Cardiology*.

[19] Desmet WJR, Adriaenssens BFM, Dens JAY (2003). Apical ballooning of the left ventricle: first series in white patients. *Heart*.

[20] Iga K, Gen H, Tomonaga G, Matsumura T, Hori K (1989). Reversible left ventricular wall motion impairment caused by pheochromocytoma. A case report. *Japanese Circulation Journal*.

